# Development of a Novel Antimicrobial Screening System Targeting the Pyoverdine-Mediated Iron Acquisition System and Xenobiotic Efflux Pumps

**DOI:** 10.3390/molecules20057790

**Published:** 2015-04-29

**Authors:** Kazuki Sato, Kenichi Ushioda, Keiji Akiba, Yoshimi Matsumoto, Hideaki Maseda, Tasuke Ando, Emiko Isogai, Taiji Nakae, Hiroshi Yoneyama

**Affiliations:** 1 Laboratory of Animal Microbiology, Department of Microbial Biotechnology, Graduate School of Agricultural Science, Tohoku University, 1-1 Tsutsumidori, Amamiya-machi, Aoba-ku, Sendai 981-8555, Japan; E-Mails: kzst129os@yahoo.co.jp (K.S.); kenichi.tonpei@gmail.com (K.U.); pyanda@sea.plala.or.jp (K.A.); tasuke@bios.tohoku.ac.jp (T.A.); emiko@bios.tohoku.ac.jp (E.I.); 2 Institute of Scientific and Industrial Research, Osaka University, Ibaraki, Osaka 567-0047, Japan; E-Mail: yoshimi@sanken.osaka-u.ac.jp; 3 Department of Biological Science and Technology, Faculty of Engineering, 2-1 Minamijyousanjima-cho, Tokushima 770-8605, Japan; E-Mail: maseda@bio.tokushima-u.ac.jp; 4 Laboratory for Antimicrobial Agents, Kitasato University, 1-15-1 Kitasato, Sagamihara 228-8555, Japan; E-Mail: nakae-tj@insti.kitasato-u.ac.jp

**Keywords:** antimicrobials, iron acquisition systems, efflux pumps, *Pseudomonas aeruginosa*

## Abstract

The iron acquisition systems in *Pseudomonas aeruginosa* are inducible in response to low-iron conditions and important for growth of this organism under iron limitation. OprM is the essential outer membrane subunit of the MexAB-OprM xenobiotic efflux pump. We designed and constructed a new model antimicrobial screening system targeting both the iron-uptake system and xenobiotic efflux pumps. The *oprM* gene was placed immediately downstream of the ferri-pyoverdine receptor gene, *fpvA*, in the host lacking chromosomal *oprM* and the expression of *oprM* was monitored by an antibiotic susceptibility test under iron depleted and replete conditions. The recombinant cells showed wild-type susceptibility to pump substrate antibiotics, e.g., aztreonam, under iron limitation and became supersusceptible to them under iron repletion, suggesting that expression of *oprM* is under control of the iron acquisition system. Upon screening of a chemical library comprising 2952 compounds using this strain, a compound—ethyl 2-(1-acetylpiperidine-4-carboxamido)-4,5,6,7-tetrahydrobenzo[*b*]thiophene-3-carboxylate—was found to enhance the efficacy of aztreonam under iron limitation, suggesting that the compound inhibits either the iron acquisition system or the MexAB-OprM efflux pump. This compound was subsequently found to inhibit the growth of wild-type cells in the presence of sublethal amounts of aztreonam, regardless of the presence or absence of dipyridyl, an iron-chelator. The compound was eventually identified to block the function of the MexAB-OprM efflux pump, showing the validity of this new method.

## 1. Introduction

Infections caused by multidrug resistant bacteria are a serious problem in hospitals and a major public health concern these days [1,2]. The discovery of new antimicrobial agents to combat such pathogens is an urgent and crucial concern [3,4,5]. One of the approaches may be to search for essential metabolic targets through genome analyses and subsequently perform target-based high-throughput *in vitro* screenings of novel lead compounds [6]. However, the agents that act upon essential metabolic targets inevitably select resistant mutants due to their strong selective pressure [7,8]. Thus, alternative approaches may be needed to tackle these issues. One such approach could be to find agents which interfere with the action of bacterial virulence factors. If anything, virulence factors are less likely to exert selective pressure on the development of resistant strains since they are virtually secondary [9,10,11]. Thus, means to control bacterial virulence open a window to search for new types of antimicrobial agents.

The iron acquisition system of bacteria is an important virulence factor for the establishment of infection [12,13]. Bacterial cells generally require micromolar orders of free iron to support their growth [12]. However, the human body sequesters iron molecules through high-affinity iron-binding proteins such as transferrin, lactoferrin, and ferritin, lowering the free-iron level to about 10^−18^ M or less [13,14]. Bacterial cells cannot grow under these iron concentrations [12,13,14]. To overcome this difficulty, bacterial cells elaborate specific iron acquisition systems under the iron-limited environment inducing high affinity iron chelator molecules and their transport systems [13,15]. The iron acquisition system in *Pseudomonas aeruginosa,* for instance, works as follows: in iron-limiting environments, the cells secrete high affinity iron chelator molecules, e.g., pyoverdine, which trap iron molecules and the iron-pyoverdine complex binds with the outer membrane receptor, FpvA. This signal may be transmitted to the inner membrane protein anti-σ factor, FpvR, and the interaction of the iron-pyoverdine-FpvA complex with the periplasmic domain of FpvR releases bound extracytoplasmic σ factors, PvdS and FpvI, promoting the transcription of the genes encoding pyoverdine biosynthetic proteins, its receptor FpvA, a virulent factor ToxA, and a protease PrpL [15,16,17]. In addition, another global regulator, Fur, negatively controls the transcription of many genes involved in the iron utilization. In iron limiting environments, extracytoplasmic σ factors such as *fpvI* and *pvdS* mediate Fur action resulting in activation of *toxA* and *prpL* [18]. The iron-uptake systems in bacteria are tightly regulated to an optimum level, since excessive intracellular iron molecules may cause the generation of oxygen radicals, which are highly toxic to the cells under aerobic conditions [12]. On the other hand, iron deficiency results in restriction of bacterial growth [12,13]. Thus, blocking the iron acquisition signal transduction circuits would be a potential means to hamper bacterial growth.

The protein PvdN, which is essential for the biogenesis of pyoverdine, is translocated to the periplasmic space across the inner membrane via the twin-arginine protein translocation machinery (Tat) [19]. Hence, blocking of the Tat protein-secretion system results in an inability to produce pyoverdine, leading to iron deficiency in *P. aeruginosa* under iron limited conditions.

The MexAB-OprM xenobiotic efflux pump consists of the inner membrane transporter MexB, the outer membrane duct protein OprM, and the periplasmic clump protein MexA [20]. The OprM protein functions as a duct in several other efflux-pump systems [20,21,22]. The pump is intrinsically expressed in the wild-type strain of *P. aeruginosa* rendering the cells resistant to multiple classes of antibiotics. The cells lacking the pump protein became supersusceptible to the pump substrate antibiotics, such as aztreonam, chloramphenicol and others [20]. Therefore, pump inhibitors represent powerful auxiliary agents to potentiate antibiotics. The aim of this study was to construct a model antimicrobial screening system targeting the iron acquisition systems, the Tat protein secretion system and/or the multidrug efflux pump(s) in *P. aeruginosa*. Then, using this newly developed system, a chemical library was screened.

## 2. Results

### 2.1. Experimental Design for Screening of Inhibitors toward Pyoverdine-Mediated Iron-Acquisition System and Xenobiotic Efflux Pumps

**Figure 1:**
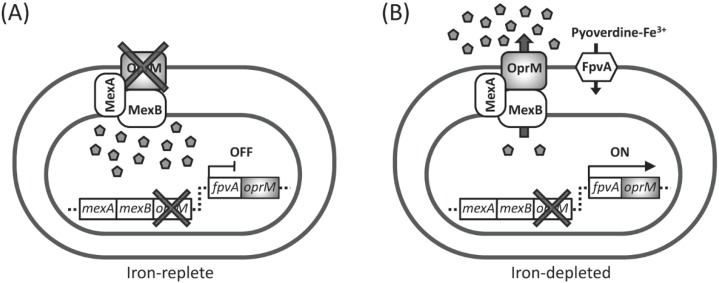
Experimental design of the screening system. The *oprM *gene is placed immediately downstream site of the *fpvA *gene of the *oprM*-deficient cell by homologous recombination. As a result, the reporter strain shows susceptibility and resistance (wild-type susceptibility) to a MexAB-OprM efflux pump substrate antibiotic (pentagons) under iron-replete (**A**) and iron-depleted (**B**) conditions, respectively, since the expression of the *oprM *gene is under the control of the *fpvA *gene.

*Pseudomonas aeruginosa* senses low iron environments and responds to them by inducing a high-affinity ferric iron chelator, pyoverdine, and its outer membrane receptor, FpvA [16,23]. If the gene coding for the outer membrane subunit of the MexAB-OprM efflux pump, *oprM*, is placed immediately downstream site of the *fpvA* gene of the *oprM*-deficient cell, expression of *oprM* will be under control of the iron acquisition system. Thus, expression of the iron acquisition system may be monitored as the resistance to the pump substrate antibiotics (Figure 1). Alternatively, pump inhibitors render the cells supersusceptible to the substrate antibiotics. Using this system, potential inhibitors of the iron-uptake system or the pump inhibitor may be screened by a simple phenotypic change of the test strain from wild-type level susceptibility to supersusceptibility in the presence of sublethal concentrations of a MexAB-OprM substrate antibiotic, e.g., aztreonam, under iron-depletion conditions (Figure 1). The reason to choose OprM subunit is that: (i) the protein collaborates with several xenobiotic exporter systems [21] and (ii) OprM-deficient cells are more susceptible to the substrate antibiotics than *mexA*- or *mexB*-deficient cells [20]. Thus, OprM serves as a pivotal efflux pump subunit suitable for a highly sensitive screening system. We constructed such a recombinant *P. aeruginosa *strain as described in the Experimental Section and the strain was designated as TNP091.

### 2.2. Antibiotic Susceptibility of the Test Strain Depends on the Extracellular Iron Levels

We next assessed the impact of the extracellular iron levels on the antibiotic susceptibility of TNP091 (Table 1). The MICs of aztreonam in TNP091 under iron-replete and depleted (in the presence of dipyridyl) conditions were 0.25 and 2.0 µg/mL, respectively, and the values were comparable to those for TNP072 lacking the *oprM* gene and TNP072 harboring pOPRM1, respectively (Table 1).

**Table 1:** MICs of aztreonam and chloramphenicol in the absence (iron-replete) or presence of dipyridyl (iron-depleted) for various strains.

Strain	Plasmid	Mic (µg/mL)			
		Aztreonam		Chloramphenicol	
		Fe-replete	Fe-depleted	Fe-replete	Fe-depleted
PAO4290 (parent)	-	4.0	4.0	32.0	32.0
TNP072 (∆*oprM*)	-	0.25	0.25	2.0	2.0
TNP072 (∆*oprM*)	pOPRM1	4.0	4.0	32.0	32.0
TNP091 (*fpvA*::*oprM*)	-	0.25	2.0	4.0	16.0
TNP092 (*fpvA*::*oprM* ∆*pvdS*)	-	0.25	0.5	4.0	4.0
TNP092 (*fpvA*::*oprM* ∆*pvdS*)	pPvdS	4.0	4.0	32.0	32.0

MICs were determined by the agar dilution method. Mueller-Hinton medium were prepared with or without 0.5 mM dipyridyl.

**Figure 2:**
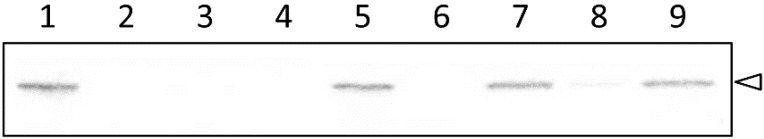
Western blot analysis of the cells grown under iron-replete and iron-depleted conditions. Cells were grown in the absence (lanes 1 to 4) or presence (lanes 5 to 9) of 0.5 mM dipyridyl and the whole cell lysate was subjected to SDS-polyacrylamide gel (10%) electrophoresis followed by visualization with an anti-OprM antibody. The position of OprM is indicated by an arrowhead. Lanes 1 and 5, PAO4290 (wild-type); lanes 2 and 6, TNP072 (Δ*oprM*); lanes 3 and 7, TNP091 (Δ*oprM*, *fpvA*::*oprM*); lanes 4 and 8, TNP092 (Δ*oprM*, *fpvA*::*oprM*, Δ*pvdS*); lane 9, TNP092 (pPvdS).

The MICs of chloramphenicol in TNP091 under iron-replete and depleted environments were 4.0 and 16 µg/mL, respectively. MICs of aztreonam and chloramphenicol in PAO4290, TNP072, and TNP072 (pOPRM1) were unchanged irrespective of the extracellular iron levels (Table 1). The wild-type susceptibility in TNP091 in the presence of dipyridyl was interpreted to mean that TNP091 cells sensed a low iron level in the milieu and this signal was conveyed to the *fpvA* promoter via an iron-responsive signal transduction pathway and induced the *oprM* gene expression. We analyzed the level of OprM expression in the presence and absence of 0.5 mM dipyridyl by the western-blotting method using an anti-OprM antibody as a probe (Figure 2). Although TNP091 produced undetectable levels of OprM under the iron-replete conditions (Figure 2, lane 3), a significant level of OprM was detected under the iron-depleted conditions (Figure 2, lane 7), confirming that OprM was induced in response to the iron-depletion.

### 2.3. The pvdS Mutation Prevents Induction of the oprM Expression

**Figure 3:**
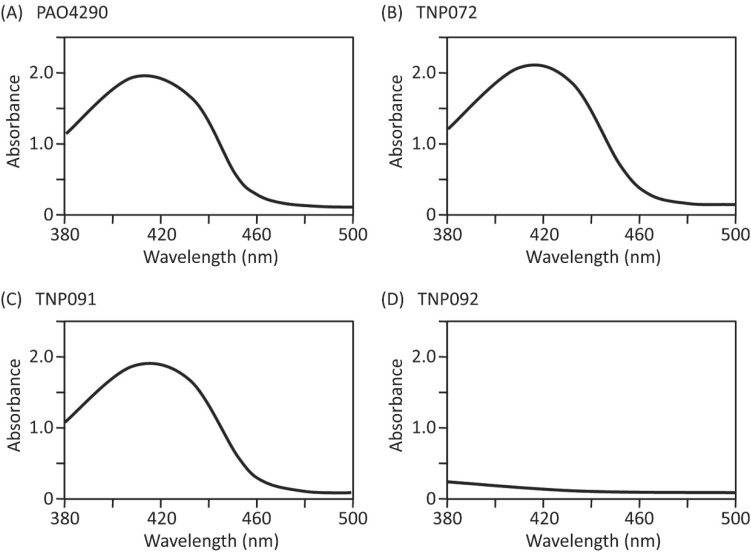
Absorption spectra of the culture supernatant. Cells were grown in casamino acid medium overnight at 37 °C under aerobic conditions and removed by the centrifugation at 13,700× *g* for 10 min. The clear supernatant fractions were scanned with a spectrophotometer. (**A**), PAO4290 (wild-type); (**B**), TNP072 (Δ*oprM*); (**C**), TNP091 (Δ*oprM*, *fpvA*::*oprM*); (**D**), TNP092 (Δ*oprM*, *fpvA*::*oprM*, Δ*pvdS*).

The above results suggested that antibiotic resistance in TNP091 in the presence of dipyridyl is most likely attributable to the induction of OprM through an iron responsive signal transduction system. To validate this assumption, we disrupted the *pvdS* gene, activation of which is essential for the induction of the *fpvA* gene and hence the *oprM* gene too [16]. First, we assessed the impact of the *pvdS* disruption on the pyoverdine production. The culture supernatant of a wild-type strain PAO4290, an *oprM*-deficient mutant TNP072, and the test strain TNP091 showed typical pyoverdine absorption spectra (Figure 3A–C), whereas that of the *pvdS* deletion mutant TNP092 produced an undetectable level of pyoverdine (Figure 3D), confirming that the iron acquisition cascade did not function at all in TNP092. The result is consistent with the previous report [16].

We next determined the MICs of antibiotics in TNP092 (Table 1). The MICs of aztreonam and chloramphenicol in TNP092 were 0.5 µg/mL and 4.0 µg/mL, respectively, under the iron-depleted conditions. The values were comparable with those obtained under the iron-replete conditions (Table 1). Consistent with this, TNP092 produced only a trace level of OprM under an iron-limiting condition (Figure 2, lane 8). To set back the *pvdS* gene into TNP092, the *pvdS* gene was cloned on a shuttle vector pMMB67HE [24] under control of *lac* promoter, pPvdS, and this plasmid was introduced into TNP092. The impact of the plasmid-borne *pvdS* expression on drug susceptibility of the transformant TNP092 (pPvdS) was tested. Under iron-depleted conditions, the cells recovered the wild-type susceptibility to both aztreonam and chloramphenicol (Table 1). A western blotting experiment revealed that the production of the OprM protein was restored in TNP092 (pPvdS) (Figure 2, lane 9). All these data supported an assumption that the wild-type susceptibility in TNP091 is attributable to the induction of OprM under iron-depleted conditions.

### 2.4. Screening of Inhibitors of the Iron-Acquisition System or the Efflux Pump in a Chemical Library

**Figure 4:**
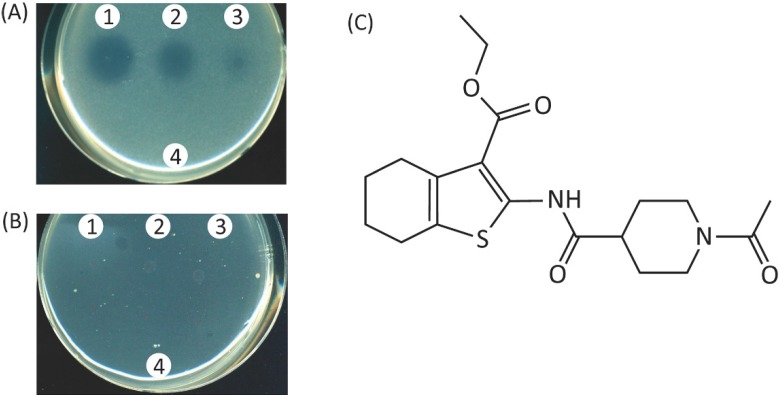
Effect of ethyl 2-(1-acetylpiperidine-4-carboxamido)-4,5,6,7-tetrahydrobenzo-[*b*]thiophene-3-carboxylate (YH001) on the growth of the test strain TNP091 (Δ*oprM*, *fpvA*::*oprM*) and chemical structure of YH001. (**A**) Liquefied Mueller-Hinton agar medium was mixed with the TNP091 cells and 1.0 µg/mL of aztreonam in the presence of 0.5 mM dipyridyl, and was solidified. Two µL of YH001 solution was spotted on the agar surface and the plate was incubated for 24 h at 37 °C. Concentrations of YH001 used were; 1, 10 mM; 2, 5 mM; 3, 2.5 mM; and 4, solvent control DMSO only. (**B**) Mueller-Hinton agar was mixed with the TNP091 cells and 1.0 µg/mL of aztreonam and was solidified. Two µL of YH001 solution was spotted on the agar surface and the plates were incubated for 24 h at 37 °C. 1, 2, 3, and 4 are the same as above. (**C**) Chemical structure of YH001.

A structurally diverse chemical compound library (n = 2952) from ChemBridge was screened for inhibitors of the iron acquisition system or a xenobiotic extrusion pump, MexAB-OprM, taking advantage of the phenotypic change of the TNP091 reporter. As expected, TNP091 showed an undetectable level of growth in the presence of 1.0 µg/mL of aztreonam in the absence of dipyridyl (Figure 4B). On the other hand, in the presence of dipyridyl, the TNP091 cells grew on the antibiotic-impregnated plate (Figure 4A). Among the chemicals in the library, one of them, ethyl 2-(1-acetyl-piperidine-4-carboxamido)-4,5,6,7-tetrahydrobenzo[*b*]thiophene-3-carboxylate (Figure 4C), showed a dose dependent growth inhibition of TNP091 in the presence of both aztreonam and dipyridyl (Figure 4A, see clear spots). This compound was designated as YH001 for convenience.

### 2.5. Characterization of the Hit-Compound

**Figure 5:**
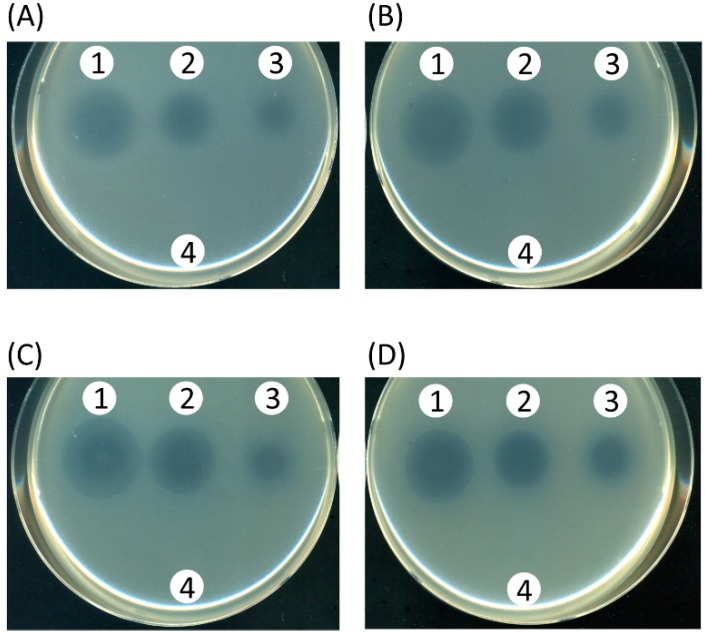
Effect of YH001 on the growth of *P. aeruginosa* producing different levels of the efflux pump in the presence of antibiotics. (**A**,**B**), a wild type strain PAO4290 producing a basal level of MexAB-OprM pump. (**C**,**D**), a strain producing an elevated level of MexAB-OprM pump, TNP30#1. Agar plates were prepared as described in the legend to Figure 4 impregnated with the following: (A) dipyridyl (0.5 mM) and aztreonam (2.0 µg/mL), (B) aztreonam (2.0 µg/mL), (C) dipyridyl (0.5 mM) and aztreonam (8.0 µg/mL), and (D) aztreonam (8.0 µg/mL). Concentrations of YH001 used were; 1, 10 mM; 2, 5 mM; 3, 2.5 mM; 4, solvent control DMSO only.

Since this screening system employed a functional MexAB-OprM efflux pump as the reporter, it is possible that the hit-compounds inhibited either any one of the iron-responsive signal transduction pathway, the efflux pump activity or the growth of the cells by non-specific cytotoxicity. To identify the target of YH001, the growth-inhibitory activity of this compound in the presence of 2.0 µg/mL of aztreonam was tested in the wild-type strain PAO4290 (MIC of aztreonam 4.0 µg/mL) in the presence and absence of 0.5 mM dipyridyl. If YH001 blocked only the iron-responsive signal transduction pathway, MexAB-OprM may be expressed from their chromosomal genes irrespective of the presence or absence of dipyridyl rendering the cells resistant to the antibiotics. However, this was not the case (Figure 5A,B).

PAO4290 was unable to grow in the presence of 2.0 µg/mL of aztreonam and YH001, regardless of the presence of dipyridyl, suggesting a possibility that compound YH001 acted on the efflux pump itself. To assure this assumption, a strain producing an elevated level of MexAB-OprM, TNP30#1 (MIC of aztreonam 25 µg/mL) was similarly tested in the presence of 8 µg/mL of aztreonam. The growth of the cells was inhibited in a similar manner to above, confirming that YH001 directly acts on the efflux pump (Figure 5C,D). As a control, we took a known pump inhibitor molecule, l-phenyl-alanyl-l-arginyl-β-naphthylamide (PAβN) [25], and determined a synergistic effect with aztreonam in PAO4290 and TNP091. The results confirmed that this inhibitor exhibited similar results to YH001 shown in Figure 4 and Figure 5 (data not shown). To see whether compound YH001 exerts non-specific cytotoxic activity, strains TNP091, PAO4290 and TNP30#1 were incubated on the aztreonam-free medium in the presence of an appropriate concentration of YH001. The result showed no detectable growth inhibitory activity (data not shown). Consistently, MIC of YH001 in TNP30#1 appeared >320 µg/mL. Taken together, all the evidence suggests that YH001 acts on the efflux pump.

**Figure 6:**
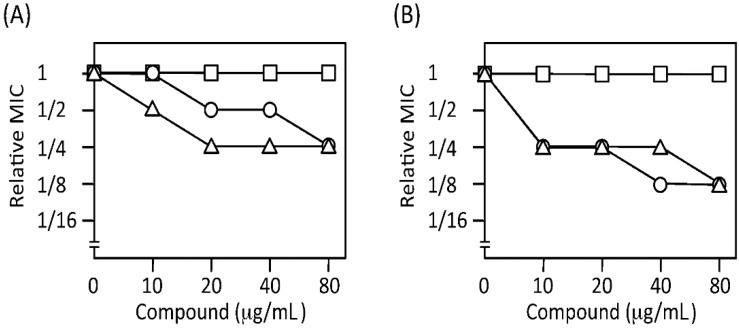
Synergistic effect of YH001 with antibiotics. Potentiation of the antibiotic effect with YH001 was assayed by the checkerboard method. The MIC values were expressed relative to that in the YH001-free medium. (**A**) *P. aeruginosa* wild type strain PAO4290. MICs of aztreonam, chloramphenicol, and imipenem in PAO4290 are 2.0, 16 and 4.0 µg/mL, respectively. (**B**) *P. aeruginosa *mutant TNP30#1 producing an elevated level of MexAB-OprM. MICs of aztreonam, chloramphenicol, and imipenem in TNP30#1 are 32, 64 and 4.0 µg/mL, respectively. Symbols: circles, aztreonam; triangles, chloramphenicol; and squares, imipenem.

Efflux pump inhibitors are known to potentiate efficacy of the efflux-pump-substrate antibiotics [26]. This possibility was tested by checkerboard assay with PAO4290 and TNP30#1. The MICs of aztreonam and chloramphenicol in PAO4290 decreased to 1/2 and 1/4 of the original MICs, respectively, in the presence of 20 µg/mL of YH001 (Figure 6A). However, that of imipenem was unchanged in the presence of even 80 µg/mL of YH001. This is attributable to the fact that imipenem is a poor substrate of the MexAB-OprM efflux pump [20]. Hence, imipenem served as an excellent control compound. Similar tendencies were observed in the pump-overproducing strain, TNP30#1 (Figure 6B). In addition, YH001 did not exert a synergistic effect with chloramphenicol in TNP072 (Δ*oprM*) and TNP076 (Δ*mexA* Δ*mexB* Δ*oprM*) as expected (data not shown). Thus, it became evident that YH001 inhibits the activity of the MexAB-OprM efflux pump in a dose dependent manner.

**Figure 7:**
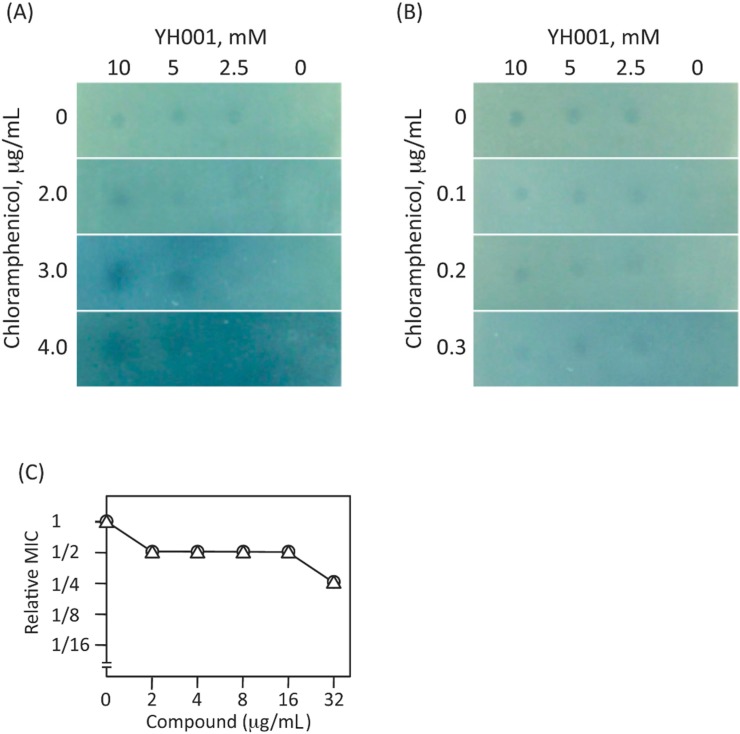
Effect of YH001 on the growth of *E. coli *strains in the presence of sub-lethal concentrations of chloramphenicol and synergistic effects of YH001 with antibiotics. (**A**) Effect of YH001 on the wild-type *E. coli* strain BW25113; (**B**) Effect of YH001 on *acrB*-deficient mutant *E. coli* JW0451 (Δ*acrB*). Agar plates were prepared mixing with the indicated concentrations of chloramphenicol and the respective bacterial cells as described in the legend to Figure 4. Two µL of YH001 solution was spotted on the surface of the agar medium and the plates were incubated at 37 °C for 24 h. (**C**) Synergistic effects of YH001 with antibiotics in the *E. coli *MG1655ΔBC (Δ*acrB*, Δ*tolC*) and *E. coli *MG1655ΔBC/pABM harboring a plasmid carrying the *mexAB-oprM *genes. MICs of antibiotics were determined by the checkerboard method. The MIC values were expressed relative to that in the YH001-free medium. MICs of aztreonam and erythromycin in the transformants are 1.0 and 32 µg/mL, respectively. Symbols: open circles, aztreonam; open triangles, erythromycin.

Since the inhibitor YH001 acts on MexAB-OprM efflux pump of *P. aeruginosa*, we asked whether this compound exerts similar activity on the major multiantibiotic efflux pump of *E. coli*, AcrAB-TolC, which is a homologue of MexAB-OprM. The growth of the wild-type *E. coli* strain BW25113 was inhibited in the presence of sublethal levels of chloramphenicol and YH001 (discernible spots in Figure 7A). Since the presence of YH001 and a sublethal concentration of chloramphenicol exerted no discernible effect on the growth of *acrB*-deficient mutant JW0451 (Figure 7B), HY001 appeared to inhibit AcrAB-TolC efflux pump as well. However, the activity of YH001 toward AcrAB-TolC seems much weaker than to MexAB-OprM. To be sure that YH001 acts on MexAB-OprM in the *E. coli* cells, the checkerboard assay was carried out using aztreonam and erythromycin in the mutant lacking *acrB *and *tolC*. The MICs of aztreonam and erythromycin were lowered to 1/2 to 1/4 in the presence of 2.0 to 32 µg/mL of YH001 (Figure 7C) compared with the value without the inhibitor, indicating clearly that the inhibitor acts directly on the MexAB-OprM efflux pump, even in the *E. coli *host.

## 3. Discussion

We have designed and constructed a new model antimicrobial compound screening system targeting the iron-acquisition system and the multidrug efflux pump(s) in *Pseudomonas aeruginosa*.

The compounds obtained from the screening by this system may have either any one of the following modes of action: (i) if the inhibitor-compound blocked the pyoverdine-synthesizing pathway, iron-pyoverdine complex may not be formed and, therefore, a σ factor, PvdS, may be kept associated with an anti-σ factor, FpvR. Thus, the test strain will be aztreonam supersusceptible; (ii) if the inhibitor blocked the function of MexAB-OprM efflux pump, the test strain will be aztreonam supersusceptible irrespective of the iron-acquisition system; (iii) if the inhibitor blocked the Tat secretion machinery, biogenesis of pyoverdine may be blocked because a periplasmic protein, PvdN, cannot be translocated to its destination [19] rendering the cells supersusceptible to aztreonam. In fact, the MICs of aztreonam and chloramphenicol in the *tatC*-deficient derivative of TNP091 dropped four-fold each compared with that in the parent cell, TNP091, under iron-depleted conditions. Since lines of evidence are accumulating that the Tat system secretes several other important virulence factors [27], blocking of this machinery likely reduces the pathogenesis of the bacteria; (iv) if the inhibitor blocked the Fur and Fur-box-mediated iron acquisition pathway, the cells will be killed, since the Fur protein is assumed to be essential for survival of *P. aeruginosa* [15].

The primary aim of the experiment was to design a new antimicrobial screening system and to construct a strain suitable for the purpose. A model system we chose was to screen potential inhibitors that block the iron acquisition systems. Advantages of using inhibitors found by this screening system over classical antibiotics may be as follows: (i) since the compounds reduce the virulence of the pathogenic bacteria but may not kill them, they are less likely to select resistant mutants; (ii) it is expected that an efflux pump inhibitor will potentiate the activity of classical antibiotics. This assumption was validated by the fact that YH001 lowered the MICs of aztreonam and chloramphenicol (Figure 6A,B).

As mentioned above, this screening system could select inhibitors of RND-type xenobiotic efflux pumps. The reason why OprM was chosen was as follows: OprM functions as a common xenobiotic discharge duct for the pump units of MexAB, MexXY, MexCD and others [20,21,22]. Therefore, if the inhibitors blocked the function of OprM, it is potentially possible to block the function of multiple species of xenobiotic efflux pumps rendering the cells susceptible to a wide variety of substrate antibiotics. To test such a possibility, the MIC of gentamicin, which is an excellent substrate of the MexXY pump, was determined in the presence of YH001. However, the MIC remained unchanged in *mexXY*-overexpressing strains in the presence and absence of YH001 (data not shown). Therefore, it is most likely that inhibitor YH001 specifically blocks the function of the MexAB pump but not the OprM exit channel. In addition, however, this compound appeared to have an inhibitory activity toward the major antibiotic efflux pump AcrAB-TolC in *E. coli* leaving a possibility that a broad spectrum inhibitor, once selected, can be extended to use in a wide range of other bacterial species. A sole drawback of this system would be that the inhibitor must be selected by negative means. However, patient and stubborn searches may open a way to combat multiantibiotic resistant pathogens.

## 4. Experimental Section

### 4.1. Bacterial Strains, Plasmids, and Growth Conditions

The bacterial strains and plasmids used are listed in Table 2. Cells were grown aerobically in L-broth containing 10 g of tryptone, 5 g of yeast extract, and 5 g of NaCl per liter (pH 7.2) at 37 °C. For the pyoverdine assay, cells were grown in medium containing 5 g per liter of casamino acids, 5 mM K_2_HPO_4_, and 1 mM MgSO_4_ under aeration at 37 °C overnight. Antibiotics used were aztreonam (Sigma-Aldrich, St. Louis, MO, USA), chloramphenicol (Wako Pure Chemicals, Tokyo, Japan), imipenem (Sigma-Aldrich) and erythromycin (Sigma-Aldrich). To generate iron-depleted conditions, dipyridyl (Sigma-Aldrich) to a final concentration of 0.5 mM was added to Mueller-Hinton agar medium.

**Table 2:** Bacterial strains and plasmids.

Strains or Plasmids	Relevant Property	Source or Reference
Strain		
*Escherichia coli* JM109	*recA*1, *endA*1, *gyrA*96, *thi*, *hsdR*17(r_k_^−^ m_k_^+^), *e*14^−^(*mcrA*^−^), *supE*44, *relA*1, Δ(*lac-proAB*)/ F'[*traD*36, *proAB*^+^, *lacI*^q^, *lac*ZΔM15]	Laboratory strain
*Escherichia coli* S17-1	RP4-2-Tc::Mu-Km::Tn7, *pro*, *res*^−^, *mod*^+^	[28]
*Escherichia coli* MG1655ΔBC	Δ*acrB*, Δ*tolC*	[29]
*Escherichia coli *BW25113	Wild type, parent of JW0451	[30]
*Escherichia coli* JW0451	Δ*acrB* derived from BW25113	[30]
*Pseudomonas aeruginosa* PAO1	wild type	Laboratory strain
*Pseudomonas aeruginosa* PAO4290	*leu-10*, *argF10*, *aph-9004*, FP-	Matsumoto collection
*Pseudomonas aeruginosa* TNP072	PAO4290 Δ*oprM*::Tc^r^	[20]
Pseudomonas aeruginosa TNP091	TNP072 *fpvA*::*oprM*	This study
*Pseudomonas aeruginosa* TNP092	TNP072 *fpvA*::*oprM,* Δ*pvdS*	This study
*Pseudomonas aeruginosa* TNP30#1	PAO4290-derived multiantibiotic resistant mutant overexpressing *mexAB-OprM* genes	[31]
**Plasmid**		
pK19mobsacB	pK19 derivative carrying RP4 *mob* region and *Bacillus subtilis sacB* gene, Km^r^	[32]
pK18mob	pK18 derivative carrying RP4 *mob* region, Km^r^	[32]
pMMB67EH	Broad host range vector, Ap^r^, IncQ	[24]
pMMB67HE	Broad host range vector, Ap^r^, IncQ	[24]
pK19FpvOprM	pK19mobsacB derivative carrying 3'-end of *fpvA* region and its downstream region in which *oprM* gene is inserted	This study
pK18mobPvdS	pK18mob derivative caring internal region of the *pvdS* gene	This study
pOPRM1	pMMB67EH derivative carrying wild type *oprM* gene, Ap^r^	[21]
pPvdS	pMMB67EH derivative carrying the *pvdS* gene	This study
pABM	pMMB67HE derivative carrying the *mexA-mexB-oprM* genes	[29]

*Abbreviations:* KM, kanamycin; TC, tetracycline; Ap, ampicillin.

### 4.2. Construction of the Recombinant Strain

To insert the *oprM* gene immediate downstream site of the *fpvA* gene in the chromosome of TNP072, a suicide vector was constructed as follows. First, a DNA fragment containing downstream region of *fpvA* was amplified using a pair of primers, FpvAdown-F and FpvAdown-R (Table 3), using the chromosomal DNA of PAO1 as the template and TaKaRa PrimeStar HS (TAKARA, Otsu, Japan) according to the manufacturer’s instructions. The resulting DNA fragments were digested with *Sma*I and *Eco*RI and subcloned into pK19mobsacB [32] pretreated with *Sma*I, *Eco*RI, and bacterial alkaline phosphatase (TOYOBO, Osaka, Japan). Next, a fragment containing *oprM* was amplified using primers, OprM-F and OprM-R (Table 3), as above, and the *Xba*I/*Sma*I-treated fragment was inserted into the above-mentioned recombinant plasmid pretreated with *Xba*I and *Sma*I. Lastly, the DNA fragment containing the 3'-end of *fpvA* gene (1428 bp) was amplified with primers, FpvA-F and FpvA-R (Table 3), as above, and the *Hin*dIII/*Xba*I-treated fragment was inserted into the *Hin*dIII and *Xba*I sites of the above *oprM*-bearing plasmid. The resulting suicide vector pK19FvpAOprM was then transferred from *E. coli* S17-1 to *P. aeruginosa* TNP072 by the filter mating method [20]. Transconjugants were selected on l-agar medium containing 12.5 µg/mL of kanamycin and 12.5 µg/mL of tetracycline at 37 °C overnight. Subsequently, the resulting transconjugants were counterselected on l-agar medium containing 10% (*w*/*v*) sucrose at 37 °C overnight. The chromosomal region of the *fpvA* gene in the resulting kanamycin-susceptible clones was analyzed by PCR using forward and reverse primers, FpvA-F and FpvAdown-R, respectively, to confirm that *oprM* was properly inserted into the downstream site of the *fpvA* gene. The strain was designated as TNP091.

To disrupt the *pvdS* gene in TNP091 cells, we first amplified internal region (419 bp) of the *pvdS* gene by TaKaRa Ex taq (TAKARA) using a primer set, PvdS-F and PvdS-R (Table 3), and the chromosomal DNA from PAO1 as the template. The resulting DNA fragments were treated with *Eco*RI and *Xba*I, and was ligated to pK18mob [32] pretreated with *Eco*RI, *Xba*I, and bacterial alkaline phosphatase, to yield pK18mobPvdS. Next, pK18mobPvdS in *E. coli* S17-1 was transferred to TNP091 by the conjugation as described above and transconjugants were selected on the medium containing 12.5 µg/mL of kanamycin and 12.5 µg/mL of tetracycline. The chromosomal region contiguous to *pvdS* was examined by PCR to confirm the insertional inactivation.

**Table 3:** Primers.

Primer	Nucleotide sequence ^a^	Tag
FpvAdown-F	5′-GGCCCCGGGGCCCGACTGCGAAAAAC-3′	*Sma*I
FpvAdown-R	5′-CGAATTCGCACGCAACTGGTGGGATAC-3′	*Eco*RI
OprM-F	5′-TGTCTAGACAAGCAGCAGGCGTCCGTC-3′	*Xba*I
OprM-R	5′-TGACCCGGGTCAAGCCTGGGGATCTTCC-3'	*Sma*I
FpvA-F	5′-ATTAAGCTTAATCCCGACACCATGCTTAC-3′	*Hin*dIII
FpvA-R	5′-GATCTAGATCAGAAGTCCCAGCGAGTG-3′	*Xba*I
PvdS-F	5′-TGAATTCACCGTACGATCCTGGTGAAG-3′	*Eco*RI
PvdS-R	5′-TGTCTAGACATGAAGTTGACCAGGGTCG-3′	*Xba*I

^a^ Restriction site tags are underlined.

### 4.3. Expression of OprM under Iron-Replete and Iron-Depleted Conditions

Fully-grown TNP091 cells in Mueller Hinton medium were diluted with a 100-fold volume of the same medium in the presence or absence of 0.5 mM dipyridyl, an iron chelator, and were continued to grow until a late-log phase. The cells were harvested by centrifugation at 9000× *g* for 10 min, washed once with phosphate-buffered saline (pH, 7.2), suspended in the same solution and the cell-free lysate was subjected to the western blot analysis.

### 4.4. Screening of a Chemical Library

The test strain TNP091 was grown in Mueller-Hinton broth at 37 °C overnight and was diluted a 100-fold with pre-warmed Mueller-Hinton agar containing 1.0 µg/mL of aztreonam and 0.5 mM dipyridyl. The agar medium was solidified in a Petri dish. Two microliters each of agents from a small molecule chemical library (ChemBridge Co., San Diego, CA, USA) dissolved in dimethyl sulfoxide (10 mM or 0.1 mM) were placed on the surface of the solid agar medium and the plates were incubated at 37 °C overnight. The compound, which inhibited the growth of the cells, was scored at 24 h of incubation.

### 4.5. Assay for the Potentiation of Antibiotic Activity by a Hit-Compound

Potentiation of the antibiotic activity by a hit-compound in the *P. aeruginosa* cells was assayed by the checkerboard method. A test strain TNP091 was grown in 2 mL of Mueller-Hinton broth containing 0.4% of KNO_3_ (*w*/*v*) at 37 °C overnight by standing culture. Five microliters of a 1000-fold diluted cell suspension (1–3 × 10^4^ cells/inoculum) were added into 2 mL of fresh Mueller-Hinton broth containing a 2-fold serially diluted antibiotic solution, to that various amounts of the compound to be tested were added. After 20 to 24 h of standing culture at 37 °C, growth of the test strain was scored by reading absorbance at 660 nm. Potentiation of the antibiotic activity by the hit-compound was also assayed in *Escherichia coli* MG1655 Δ*acrB*Δ*tolC* producing plasmid-borne MexAB-OprM by the microdilution method in a 96-well plate as described previously [29]. The synergistic effect was calculated by dividing an MIC of antibiotic in the presence of the hit-compound by that without the compound.

### 4.6. Other Techniques

The MIC of antibiotics was determined by the agar dilution method using Mueller-Hinton agar (Becton, Dickinson and Company, Sparks, MD, USA) [33]. Pyoverdine was quantified by measuring the absorption spectra from 380 nm to 500 nm of the culture supernatants [33]. SDS-PAGE was carried out by the method of Laemmli [34]. To determine the expression levels of OprM, the whole cell lysates were subjected to SDS-PAGE followed by blotting onto a polyvinylidene fluoride membrane (Millipore, Billerica, MA, USA). OprM was visualized by probing with an anti-OprM antibody using an alkaline phosphatase-conjugated secondary antibody [20]. The protein concentration was quantified by the method of Lowry *et al.* [35]. The nucleotide sequence was determined by the dideoxy chain termination method [36].
